# Phonon Conduction in Silicon Nanobeam Labyrinths

**DOI:** 10.1038/s41598-017-06479-3

**Published:** 2017-07-24

**Authors:** Woosung Park, Giuseppe Romano, Ethan C. Ahn, Takashi Kodama, Joonsuk Park, Michael T. Barako, Joon Sohn, Soo Jin Kim, Jungwan Cho, Amy M. Marconnet, Mehdi Asheghi, Alexie M. Kolpak, Kenneth E. Goodson

**Affiliations:** 1 Department of Mechanical Engineering, Stanford University, Stanford, CA 94305 USA; 2 Department of Mechanical Engineering, Massachusetts Institute of Technology, Cambridge, MA 02139 USA; 3 Department of Electrical Engineering, Stanford University, Stanford, CA 94305 USA; 4 Department of Electrical and Computer Engineering, The University of Texas at San Antonio, San Antonio, TX 78249 USA; 5 Department of Materials Science and Engineering, Stanford University, Stanford, CA 94305 USA; 6 Geballe Laboratory for Advanced Materials, Stanford University, Stanford, CA 94305 USA; 7 Department of Mechanical Engineering, Kyung Hee University, Yongin-si, 446-701 South Korea; 8 School of Mechanical Engineering, Purdue University, West Lafayette, Indiana 47907 USA

## Abstract

Here we study single-crystalline silicon nanobeams having 470 nm width and 80 nm thickness cross section, where we produce tortuous thermal paths (*i*.*e*. labyrinths) by introducing slits to control the impact of the unobstructed “line-of-sight” (LOS) between the heat source and heat sink. The labyrinths range from straight nanobeams with a complete LOS along the entire length to nanobeams in which the LOS ranges from partially to entirely blocked by introducing slits, *s* = 95, 195, 245, 295 and 395 nm. The measured thermal conductivity of the samples decreases monotonically from ~47 W m^−1^ K^−1^ for straight beam to ~31 W m^−1^ K^−1^ for slit width of 395 nm. A model prediction through a combination of the Boltzmann transport equation and *ab initio* calculations shows an excellent agreement with the experimental data to within ~8%. The model prediction for the most tortuous path (*s* = 395 nm) is reduced by ~14% compared to a straight beam of equivalent cross section. This study suggests that LOS is an important metric for characterizing and interpreting phonon propagation in nanostructures.

## Introduction

Understanding phonon transport is crucial to engineering novel nanoscale systems, such as thermal energy storage^1–3^, heat-assisted memory^4, 5^, sensors^6, 7^, and integrated nanoelectronics^8, 9^. Previous studies of phonons in single crystalline silicon have established an increasingly comprehensive microscopic model of how phonons interact with introduced nanoscale features and boundaries^10^. In many nanostructures, *e*.*g*., nanobeams and nanowires, a key geometric feature is an unobstructed line-of-sight (LOS) for propagation through the medium. In these nanostructures, the boundaries are aligned along the dominant direction of heat flow and the LOS along the dominant direction of heat flow is typically much longer than the phonon mean free paths (MFPs)^11–15^. For the case of nanostructures with boundaries or interfaces that are oriented normal to the direction of heat flow, much progress has been made for superlattices and related nanostructures and has illustrated the impact of ballistic and even coherent transport^16–20^. However, the interplay of these two types of interfaces in practical structures remains both insufficiently understood and a barrier to the effective simulation of many practical and applied nanostructures.

In complex nanostructures, phonon scattering off longitudinal boundaries and obstructions (*i*.*e*. scattering sites oriented normal to the net heat flux vector) reduces the thermal conductivity. For example, thin films containing a high-density of nanoscale holes show a dramatic reduction in the in-plane thermal conductivity^13, 21–23^. These silicon structures contain a limited LOS path between adjacent holes in a given row in the direction of heat flow and a continuous LOS path between neighboring rows of holes in the direction of heat flow^13, 21, 24^. The contribution of the limited LOS path to thermal conductivity is not clearly understood, which makes it difficult to determine the impacts of the placement^25–27^, separation^13^ and size^28^ of holes on thermal transport. While previous work has explored the impact of forward boundary scattering^29^, the relevant physics is still elusive for phonons scattering off forward obstructions.

**Figure 1:**
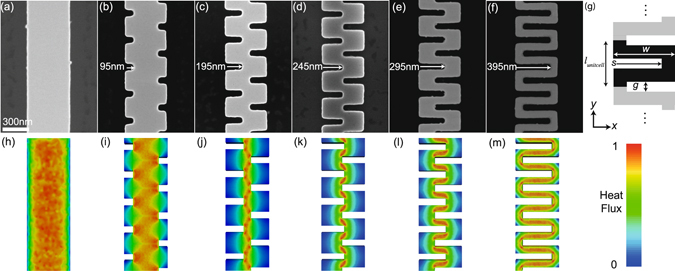
(**a**–**f**) Magnified view of nanostructured samples with various widths of slits up to 395 nm as marked on the images. (**g**) Schematic of a sample unit cell defining critical geometric dimensions. (**h**–**m**) Heat flux magnitude of the samples. The heat flux is obtained by solving the Boltzmann transport equation. The heat flux is normalized per sample and shown in the legend.

Here we demonstrate the impact of the limited LOS in silicon nanobeams that feature deliberately engineered forward obstructions along the direction of a net heat flow. We refer to these tortuous beams as labyrinths, which originates from the maze structure designed to hinder the escape of the mythological Minotaur. In nanostructured labyrinths, ballistic phonons are spatially confined by the boundaries. The baseline reference is a thin (thickness *t* = 80 nm) and narrow (width *w* = 470 nm) silicon beam that is 10 *µ*m long, and phonons can travel unimpeded along the major axis of the beam. We introduce obstructions along this beam axis by adding offset slits to disrupt the thermal transport path (see Fig. 1). The slits are cut into the beam from the edge to increase the phonon scattering rate and further limit the maximum phonon axial-travel length. This effectively eliminates any continuous LOS at the point when the adjacent slits overlap each other. We fabricate six different samples, including a reference silicon nanobeam without slits and five nanobeams with slits that vary in width *s* from 95 nm to 395 nm, as shown in Fig. 1(a–f).

### Experiments

**Figure 2:**
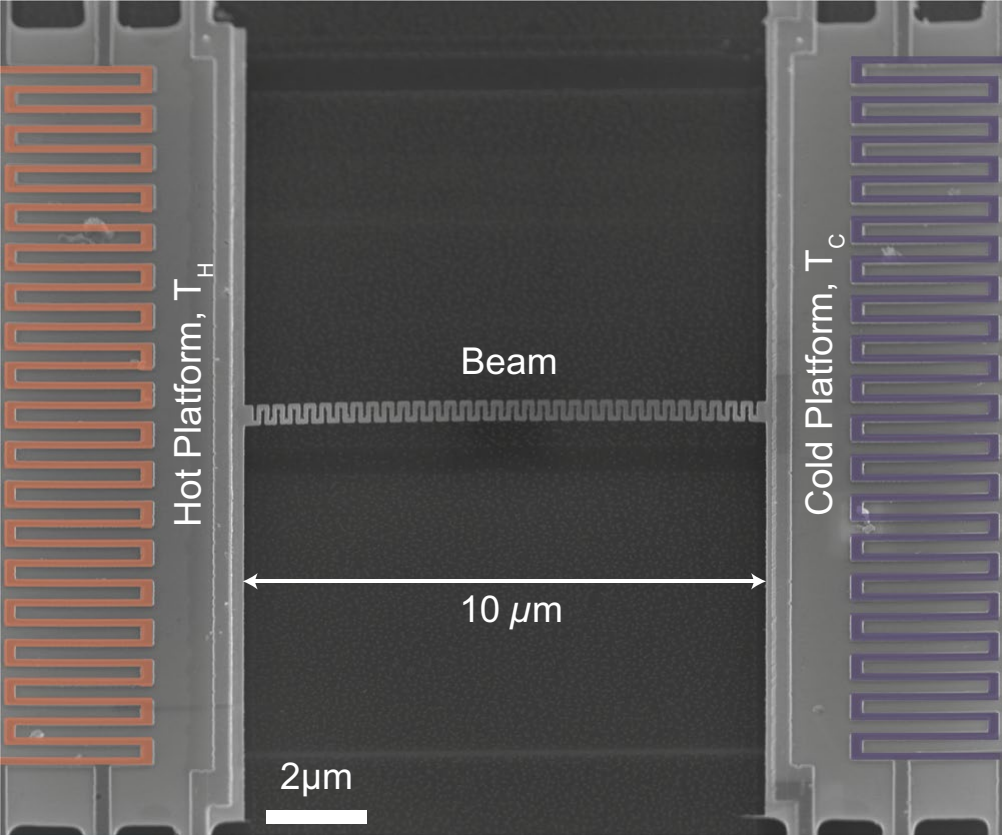
Scanning electron microscope (SEM) images of the measurement structure with false-colored serpentine heater/thermometers. The SEM image is rotated by 45 degrees about the major beam axis. The residual shadow below the beam confirms that it is fully suspended over the substrate.

The experimental structures are patterned between hot and cold platforms, and are suspended for thermal characterization as shown in Fig. 2. Heat is generated in one island and conducts through the sample and the supporting beams, and temperature is measured in each platform to measure the thermal conductance of the sample^30, 31^. We extract the thermal conductivity of the samples by fitting the measured thermal conductance of the samples to the numerically calculated values using a finite element method (solved using COMSOL). The numerical simulation solves a three-dimensional heat equation that accounts for the volumetric change in thermal conductance between samples, assuming constant and homogeneous properties within the entire beams. We note that the finite element method thereby provides a reference, from the diffusive limit, for the impacts of geometry on transport, and the thermal conductivity allows us to examine departures from diffusive theory separately from geometry. All of the measurements are performed under vacuum to minimize convective heat losses from the beams, and the heat loss through radiation is considered negligible compared to the heat conduction through the samples at all temperatures in the study^31^. The thermal interfacial resistances between the sample and the platforms are negligible since the devices are monolithically fabricated, which minimizes contact resistances^13, 22, 32^. The uncertainty is primarily due to the measurement of the sample dimensions, and the uncertainty propagation is available in Supplementary Information.

## Results

For data interpretation, it is helpful to categorically divide the samples into two categories, those having a continuous LOS (Fig. 1(a–c)) and those with a blocked LOS (Fig. 1(d–f)). In the continuous LOS category, an open channel exists through the center of the major beam axis between heat source and heat sink. In the blocked LOS category, the maximum phonon travel distance is dictated by the forward spacing between the boundaries, where the maximum phonon travel distance is comparable to phonon MFPs, (*i*.*e*. partially ballistic transport). The heat flux maps are obtained by solving the Boltzmann transport equation and clearly show the different LOS channels as seen Fig. 1(h–m). We also define the coordinate system in Fig. 1(g), where the *x*- and *y*-axes are defined with respect to the axial direction of the beam.

**Figure 3:**
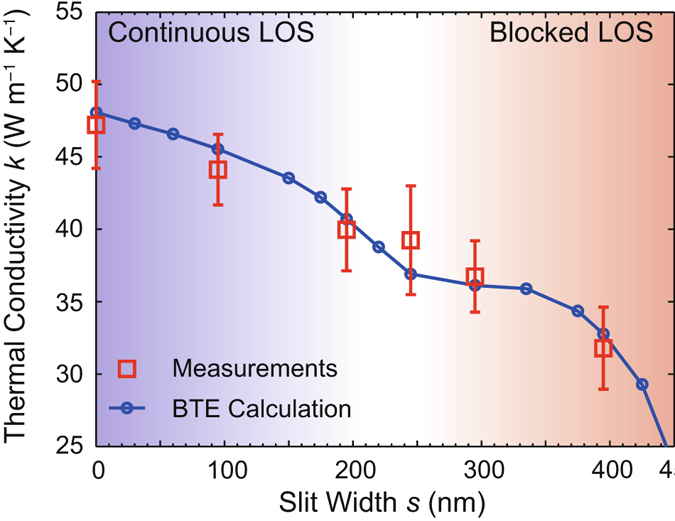
Thermal conductivity *k* of the nanostructured silicon samples with varying slit width *s*. The blue solid line is a model prediction that is obtained by solving Boltzmann Transport equation.

As slit width *s* increases, the phonon transport reduction mechanism changes from a constricted but continuous LOS to a forcibly tortuous heat flow and fully blocked LOS. The measured thermal conductivity of the samples decreases monotonically from ~47 W m^−1^ K^−1^ to ~31 W m^−1^ K^−1^ with increasing *s* as shown in Fig. 3. The model prediction described below shows a sharp change in thermal conductivity at transition from continuous LOS along the entire beam to blocked LOS by geometry (*i*.*e*., *s* = 235 nm). A combination of experimental results and the modeling prediction indicates that phonon suppression mechanisms between the continuous and blocked LOS categories are dissimilar. In the continuous LOS samples, the central open channel through the beams is the primary heat transport path as seen in Fig. 1(h–j). With decreasing width of the LOS, heat flux through the LOS channel is continuously reduced, and phonon traveling in *x*-direction is locally induced. Possible phonon trajectories can be grouped into two cases: phonons propagating through the central LOS and phonons experiencing additional boundary scattering between adjacent slits in *y*-axis. A fraction of these two cases is controlled with the slit width *s*, and this modulation results in monotonically reduced thermal conductivity with increasing *s*. In the blocked LOS samples, the LOS is limited by geometry, and the tortuous thermal path forces heat flow to change direction between *x*- and *y*-axis. The change of heat flow along the thermal path is shown in Fig. 1(k–m). In this regime, phonon propagation experience additional scattering from boundaries normal to the direction of predominant heat flow. The reduction in thermal transport is due to both spatially confined cross-section and the limited longitudinal dimension along dominant heat flow. The samples in the blocked LOS can be considered as a series of alternating thermal resistors, ones aligned in *x* direction and the other aligned in *y* direction. With decreasing slit width *s*, the cross-section of the resistors aligned with in *y*-axis is continuously reduced, and this transverse constriction further suppresses thermal conductivity in the blocked LOS. Also, each resistor has a limited LOS in a direction of heat flow, and this limited length scale impacts on phonon propagation through the beam.

We investigate the impact of limited LOS in a direction of the dominant heat flow by comparing the tortuous beam with *s* = 395 nm and a straight beam with an equivalent cross-section and length. The tortuous beam with *s* = 395 nm has a cross-sectional area of 75 nm × 80 nm cross-sectional area throughout the samples. The thermal conductivity for the straight beam with a cross-section of 75 nm × 80 nm is predicted to be 37.6 W m^−1^ K^−1^ using the model as described below. The tortuous beam with *s* = 395 nm are estimated to be 33.1 W m^−1^ K^−1^ and ~31 W m^−1^ K^−1^ from a model prediction and experiments results, respectively. Both estimations show ~14% and ~18%, respectively, smaller conductivity values than the prediction for the straight beam despite sharing the same cross-sectional area. This discrepancy of the thermal conductivity between these two geometries isolates the contribution from the limited LOS. Compared to the straight beam with *w* = 75 nm, the tortuous beam with *s* = 395 nm has 130 additional scattering corner sites. Following diffuse scattering from the boundary within the structures, these phonons scatter in the opposite direction of the dominant heat flow, resulting in a reduction of MFP^22^.

## Discussion

We model thermal transport in the nanostructured samples by solving the steady-state, phonon Boltzmann transport equation under the relaxation time approximation^33^. The thermal conductivity is described using a combination of bulk MFPs and the suppression function as given by^34^

1 $$k={\int }_{0}^{\infty }{\int }^{}{S}_{{\rm{\Lambda }}}({\rm{\Lambda }},{\rm{\Omega }})f({\rm{\Lambda }})d{\rm{\Omega }}d{\rm{\Lambda }}$$

where *f* is the differential MFP distributions for bulk silicon, *S*_Λ_ is the directional phonon suppression function, Ω is a solid angle, and Λ is an integration variable for MFP of bulk silicon. The suppression function *S*_Λ_(Λ, Ω) describes the suppression of heat carried by phonons with MFP Λ and direction Ω with respect to Fourier’s law prediction^24^. The directional suppression function is computed by the Boltzmann transport equation as described in Method section. The calculation in the present work uses MFPs for a bulk medium as input, where the bulk MFP spectra is obtained from *ab initio* calculations^35^. The calculation of the Boltzmann transport equation predicts thermal conductivity with varying *s*, and the prediction agrees with experimental results to within ~8%. This indicates that phonon suppression mechanisms are captured by the calculation of the Boltzmann transport equation, validating the use of diffuse scattering boundary conditions at room temperature^36^.

**Figure 4:**
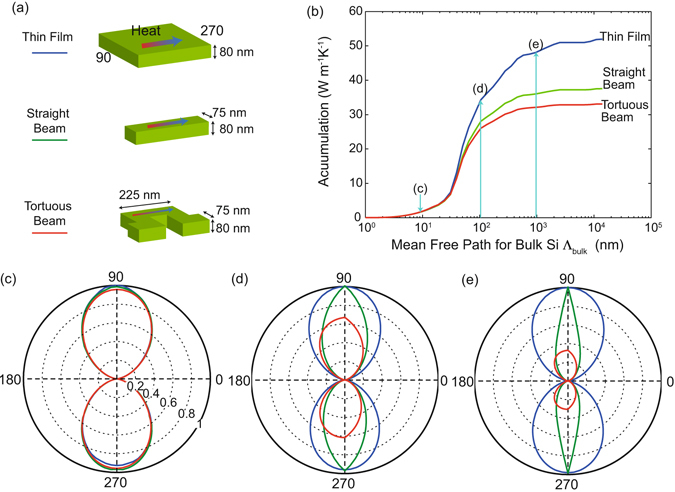
(**a**) Schematics for simulations, which includes a thin film, a straight beam, and a tortuous beam. (**b**) Thermal conductivity accumulation with bulk MFP Λ_*bulk*_. The blue arrow indicates the MFPs, where a direction suppression function is shown at marked figures (**c**,**d**). Direction suppression function for (**c**) Λ_*bulk*_ = ~9 nm, (**d**) Λ_*bulk*_ = ~100 nm, and (**e**) Λ_*bulk*_ = ~900 nm. Heat flows from *ϕ* = 90 to *ϕ* = 270. These suppression functions are normalized with the value of a thin film, and the relative magnitude is shared among (**c**–**e**).

Based on the calculation of Boltzmann transport equation, we calculate the accumulated thermal conductivity with bulk MFPs to obtain a MFP-specific understanding of boundary scattering as a function of characteristic length scales. We compare three different cases: a thin film with thickness *t* = 80 nm, a straight beam with *t* = 80 nm and width *w* = 75 nm, and a tortuous beam with *t* = 80 nm, *w* = 75 nm, and *s* = 395 nm as shown in Fig. 4(a). The comparison among these cases shows the individual impact of respective dimensions: thickness *t*, width *w*, and the LOS. The accumulated thermal conductivity is

2 $$k({{\rm{\Lambda }}}_{bulk})={\int }_{0}^{{{\rm{\Lambda }}}_{bulk}}{\int }^{}{S}_{{\rm{\Lambda }}}({\rm{\Lambda }},{\rm{\Omega }})f({\rm{\Lambda }})d{\rm{\Omega }}d{\rm{\Lambda }}$$

where Λ_*bulk*_ is phonon MFPs of bulk silicon. The function for thin film is calculated from the aforementioned model with a periodic boundary condition along *x*-axis. The accumulation functions show the impact of limiting dimensions, thickness *t*, width *w*, and the limited LOS in the dominant heat flow direction. The accumulation functions are reduced from bulk to the thin film, from the thin film to the straight beam, from the straight beam to the tortuous beam with *s* = 395 nm, and these corresponds to the impact of thickness, width, and the length of LOS, respectively. The accumulation functions for the thin film and the beam start to deviate from those for the bulk and the thin film, respectively, near *t*/3 = ~25 nm. The accumulation function for the sample with *s* = 395 nm shows suppression from a third longitudinal length, ~ 75 nm. The accumulation clearly shows the transition regime where boundary scattering becomes significant, and it is found that the limited LOS contributes to the reduction in thermal conductivity by suppressing long MFPs phonons.

To further investigate the impact of limited LOS, we use the directional suppression function, which provides angular information of suppressed heat flux for a given geometry. Since the samples are planar structures, we plot the directional suppression function on *x*-*y* plane, and the suppression function is defined as,

3 $${{\rm{S}}}_{{\rm{\Lambda }}}({\rm{\Lambda }},\varphi )=\frac{1}{2}{\int }_{0}^{\pi }{S}_{{\rm{\Lambda }}}({\rm{\Lambda }},\theta ,\varphi )sin(\theta )d\theta $$

where the function is integrated over the azimuthal angle *θ*. We note that a conventional suppression function S_Λ_ is proportional to only the magnitude of heat flow, and the integration of S_Λ_(Λ, *ϕ*) over *ϕ* provides the ratio of the MFP dependent heat flow to the counterpart of the bulk. The directional suppression function S_Λ_ is comprised of two symmetric lobes pointing toward +y and −y directions, and S_Λ_ vanhishes along the *x*-axis since there is no net heat flux for *ϕ* = 0 and 180 degrees. The suppression functions are compared at MFP = ~9 nm, ~100 nm, and ~900 nm, as shown in Fig. 4(b–d). The MFPs are chosen to represent three cases, internal scattering process dominant region (diffusive regime), the transition from internal scattering to boundary scattering (quasi-ballistic regime), and boundary scattering dominant region (nearly ballistic regime). While the suppression functions for the case of MFP = ~9 nm are overlapped among all the geometries due to the relative dominance of internal scattering, the suppression functions for MFP = ~900 nm clearly shows the impact of each dimension. The suppression function is normalized with the case of thin films to show the individual impact of each limiting dimension. For the cases of MFP ~100 nm and ~900 nm, the lobe for the straight beam is narrowed in compared to that of thin films, and this change indicates the impact of the limited width, *w* = 75 nm. The longitudinal heat flow of the tortuous beam with *s* = 395 nm is further suppressed relative to the straight beams due to forward boundaries, and this impact is displayed in reduction of heat flux along *y*-axis for MFP ~100 nm and ~900 nm. The suppression function analysis suggests that the impact of limited LOS becomes relevant when the distance that phonons travel is comparable to the MFP. With diffuse boundary scattering, this length scale can be considered as a limiting dimension, such as the diameter of a nanowire. These findings suggest that controlling the longitudinal dimension along heat flow can manipulate phonon propagation, and the relative contribution of limited LOS to thermal conductivity is important because it reduces the length of longitudinal free path.

### Summary

The combination of experiments and the Boltzmann transport equation calculations provides a quantitative description of the impact of conduction blocking the LOS channels along the direction of dominant heat flow. Tortuous thermal paths suppress the effective phonon MFP due to longitudinal features, and the suppression in heat flux is stronger than that from the cross-sectional confinement alone. This provides guidance to the design of nanostructures that achieve a lower limit to thermal conductivity. The suppression of phonon propagation using this limited LOS approach can be useful for thermoelectric applications. While previous studies of nanostructured thermoelectric materials show reduced thermal conductivity by increasing scattering events, such spatial confinement also causes simultaneous reduction of electrical conductivity, which degrades the figure or merit in applications such as thermoelectrics^14, 21, 37^. Engineering thermal paths enables manipulation of energy carriers by suppressing phonon thermal conductivity more efficiently with constant cross-sectional area, which aids in decoupling phonons and electrons into ballistic and diffusive regimes, respectively. This work improves the understanding of phonon conduction in modern devices where both ballistic and diffusive phonon transports are present since the length scales of all three spatial dimensions are comparable to the phonon MFP, such as in fin field effect transistors (FinFETs).

## Methods

### Sample Fabrication

We fabricate silicon nanobeams on silicon-on-insulator (SOI) wafers (Soitec Inc.) comprised of 340 nm-thick silicon device layer and a 1 *µ*m-thick buried oxide (BOX) layer and patterned using electron beam lithography. First, the device layer is thinned down to ~80 nm via thermal oxidation and subsequent removal of the oxide using buffered oxide etchant (BOE). The reduced thickness of a device layer is measured using an optical ellipsometer and confirmed using transmission electron microscopy (TEM) imaging. We deposit ~25 nm-thick Al_2_O_3_ using an atomic layer deposition (ALD) for electrical passivation between the metal contacts and the device layer, and the 10 *µ*m by 20 *µ*m sample region window between the two islands is opened by etching Al_2_O_3_ using a combination of reactive ion etching (RIE) and wet etching using BOE. We pattern the nanobeams and slits using electron beam lithography, and RIE is used to remove the silicon to form the slits. The metal heater lines on the islands and the contact pads for electrical access are patterned using a combination of electron beam- and photo-lithography, and a lift-off process is followed after deposition of ~5 nm-thick Cr and ~40 nm-thick Pt using electron beam metal evaporation. The surrounding area of the islands and legs are etched using RIE, and they are suspended using gaseous hydrogen fluoride (HF) etching. All dimensions are measured using scanning electron microscopy (SEM) after fabrication and TEM is used to measure the thickness of silicon beam.

### Boltzmann Transport Equation Calculation

The computational domain is a unit-cell of a beam as shown in Fig. 1(g), and the unit cell is a beam of width *w* = 470 nm, length *l*_unitcell_ = 300 nm and thickness *t* = 80 nm. We solve the Boltzmann transport equation (BTE) with varying s from 0 to 395 nm as denoted in Fig. 1(a–f). Periodic boundary conditions are applied on the boundaries along the y-axis with a temperature difference of Δ*T* = 1 K, and diffuse boundary scattering is assumed for the other boundaries. The BTE solver uses the MFP spectra as only input^38^, which is computed by *ab initio* calculations^35^. The BTE is solved in space by the finite-volume method^39^ while the angular space is discretized by means of the discrete ordinate method. Specifically, we achieve convergence in the thermal conductivity when the BTE is solved for 100 MFPs, 96 polar angles (defined on the x-y plane) and 24 azimuthal angles. For the MFPs smaller than ~2 nm, we employ a diffusive equation, which enables accurate simulations and seamless integration with the BTE with a reasonable spatial discretization.

## Electronic supplementary material

Supplementary Information
