# Supplementary material for: Phonon Conduction in Silicon Nanobeam Labyrinths
Source: Sci Rep. 2017 Jul 24;7:6233. doi: 10.1038/s41598-017-06479-3 (PMC5524879; doi:10.1038/s41598-017-06479-3)
Supplement: Supplementary file 1 — Supplementary Information [file 41598_2017_6479_MOESM1_ESM.pdf]

[Supplementary Information]

Phonon Conduction in Silicon Nanobeam Labyrinths

Woosung Park[1], Giuseppe Romano[2], Ethan C. Ahn[3,4], Takashi Kodama[1], Joonsuk Park[5], Michael T. Barako[1], Joon Sohn[3], Soo Jin Kim[6], Jungwan Cho[1,7], Amy M. Marconnet[8], Mehdi Asheghi[1], Alexie M. Kolpak[2], and Kenneth E. Goodson[1,*]

[1]Stanford University, Department of Mechanical Engineering, Stanford, CA, 94305, USA

[2] Massachusetts Institute of Technology, Department of Mechanical Engineering, Cambridge, MA, 02139, USA

[3]Stanford University, Department of Electrical Engineering, Stanford, CA, 94305, USA

[4]The University of Texas at San Antonio, Department of Electrical and Computer Engineering, San Antonio, TX 78249, USA

[5]Stanford University, Department of Materials Science and Engineering, Stanford, CA, 94305, USA

[6]Stanford University, Geballe Laboratory for Advanced Materials, Stanford, CA, 94305, USA

[7]Kyung Hee University, Department of Mechanical Engineering, Yongin-si, 446-701, South Korea

[8]Purdue University, School of Mechanical Engineering, West Lafayette, Indiana 47907, USA

Uncertainty Analysis

We quantify the measurement uncertainty using root mean square average for the contribution of each component as following

$$\left.\frac{\Delta k}{k}\right|_{Total} = \sqrt{\left(\left.\frac{\Delta k}{k}\right|_{Var.1}\right)^2 + \left(\left.\frac{\Delta k}{k}\right|_{Var.2}\right)^2 \cdots} \quad \text{(S1)}$$

where k is thermal conductivity and Var indicates sources of uncertainty. The error is predominantly attributed to the uncertainty in dimensions of the samples, which is inherently caused by tolerances in nanofabrication and scanning electron microscope (SEM) measurements. We summarize the uncertainty in dimensions and its propagation to thermal conductivity in Table S1. The measurement error caused by uncertainty in system dimensions for all samples is less than ~10% of the thermal conductivity.

Table S1. Uncertainty Analysis

Samples	k ($Wm^{-1}K^{-1}$)	w (±5 nm) Error (%)	s (±5 nm) Error (%)	g (±5 nm) Error (%)	t (±5 nm) Error (%)	Total Error (%)
s = 0 nm	47.2	1.1	-	-	2.4	6.3
s = 95 nm	44.1	0.7	2.5	0.1	2.4	5.5
s = 195 nm	40.0	0.7	5.3	1.8	2.4	7.1
s = 245 nm	39.2	1.2	6.4	3.7	2.4	9.6
s = 295 nm	36.7	0.2	3.9	4.3	2.4	6.7
s = 395 nm	31.8	0.1	3.1	5.0	2.4	8.9
